# Effects of Abdominal Rotation on Jump Performance in the Ant *Gigantiops destructor* (Hymenoptera, Formicidae)

**DOI:** 10.1093/iob/obz033

**Published:** 2019-12-18

**Authors:** Dajia Ye, Joshua C Gibson, Andrew V Suarez

**Affiliations:** 1 School of Integrative Biology, University of Illinois at Urbana–Champaign, Urbana, IL 61801, USA; 2 Department of Biology, University of Pennsylvania, Philadelphia, PA 19104, USA; 3 Department of Entomology, University of Illinois at Urbana–Champaign, Urbana, IL 61801, USA; 4 Department of Evolution, Ecology and Behavior, University of Illinois at Urbana–Champaign, Urbana, IL 61801, USA

## Abstract

Jumping is an important form of locomotion, and animals employ a variety of mechanisms to increase jump performance. While jumping is common in insects generally, the ability to jump is rare among ants. An exception is the Neotropical ant *Gigantiops destructor* (Fabricius 1804) which is well known for jumping to capture prey or escape threats. Notably, this ant begins a jump by rotating its abdomen forward as it takes off from the ground. We tested the hypotheses that abdominal rotation is used to either provide thrust during takeoff or to stabilize rotational momentum during the initial airborne phase of the jump. We used high speed videography to characterize jumping performance of *G. destructor* workers jumping between two platforms. We then anesthetized the ants and used glue to prevent their abdomens from rotating during subsequent jumps, again characterizing jump performance after restraining the abdomen in this manner. Our results support the hypothesis that abdominal rotation provides additional thrust as the maximum distance, maximum height, and takeoff velocity of jumps were reduced by restricting the movement of the abdomen compared with the jumps of unmanipulated and control treatment ants. In contrast, the rotational stability of the ants while airborne did not appear to be affected. Changes in leg movements of restrained ants while airborne suggest that stability may be retained by using the legs to compensate for changes in the distribution of mass during jumps. This hypothesis warrants investigation in future studies on the jump kinematics of ants or other insects.

## Synopsis

Spanish: Efectos de la rotación abdominal en el desempeño del salto de la hormiga *Gigantiops destructor* (Hymenoptera, Formicidae) El salto es una forma importante de locomoción y muchos animales utilizan diversidad de mecanismos al saltar para mejorar su desempeño. A pesar de que el salto es común en insectos, en general, las hormigas presentan una habilidad limitada. La hormiga neotropical *Gigantiops destructor* (Fabricius 1804) es una excepción, y utiliza el salto para capturar presas o escapar de potenciales amenazas. Esta especie empieza el salto rotando el abdomen anteriormente al impulsarse desde el suelo. Se evaluaron las hipótesis que la rotación abdominal se usa tanto para la proporción de empuje durante el impulso, así como en la estabilización de la cantidad de movimiento rotacional durante la fase inicial del salto mientras se encuentra en el aire. Se usó videografía de alta velocidad para caracterizar el desempeño del salto entre dos plataformas. Posteriormente, un grupo de hormigas fueron anestesiadas, y con el uso de pegamento, se restringió el movimiento del abdomen para evitar la rotación de éstos en la subsecuente caracterización del desempeño al saltar. Los resultados apoyan la hipótesis que la rotación abdominal proporciona impulso adicional. La distancia máxima, el peso máximo y la velocidad del impulso durante el salto fueron reducidos cuando el abdomen está fijo comparados con los saltos de las hormigas que no sufrieron manipulación y las que se usaron en el tratamiento control. En contraste, no hubo evidencia que la estabilidad de rotación de las hormigas mientras se encontraban en el aire fuera afectada. Las hormigas con abdómenes fijos presentaron cambios en el movimiento de las patas que sugieren que la estabilidad se puede mantener al usar las patas y compensar la distribución de la masa durante el salto. Esta hipótesis justifica futuros estudios evaluando la cinemática del salto en hormigas y otros grupos de insectos. Translated to Spanish by Rafael Achury (rafaelachury@gmail.com)

French: Effet de la rotation abdominal sur les performances du saut chez la fourmi *Gigantiops destructor* (Hymenoptera, Formicidae) Sauter est une importante forme de locomotion, et les animaux utilisent une diversité de mécanismes pour améliorer les performances de leurs sauts. Meme si sauter est commun chez les insectes en général, la capacité de sauter est rare chez les fourmis. La fourmi néotropicale *Gigantiops destructor* (Fabricius 1804) est une exception, elle est reconnue pour sauter sur ces proies ou pour s’échapper des menaces. Singulièrement, cette fourmi commence un saut par une rotation de son abdomen vers l’avant au moment de décoller du sol. Nous avons testé l’hypothèse que la rotation abdominale est utilisée pour soit générer une poussée au décollage, soit stabiliser l’élan rotatif pendant la phase aérienne initiale du saut. Nous avons utilisé l’enregistrement vidéo de grande vitesse pour caractériser la performance du saut des ouvrières *G. destructor* entre deux plateformes. Ensuite, nous avons anesthesié les fourmis et utilisé de la colle pour empêcher leurs abdomens de pivoter durant les prochains sauts, pour de nouveau caractériser la performance du saut suite à la restriction dudit abdomen de cette manière. Nos résultats soutiennent l’hypothèse que la rotation de l’abdomen entraine une poussée supplementaire vu que la distance maximale, la hauteur maximale et la vitesse de décollage des sauts sont réduites par la restriction du mouvement de l’abodmen comparer aux sauts des fourmis non manipulées du groupe témoin. Au contraire, la stabilité rotative des fourmis en phase aérienne ne semble pas être affectée. Les changements dans le mouvement des pattes des fourmis restraintes suggèrent que la stabilité peut être conservée en utilisant les pattes pour compenser les variations de la distribution de la masse pendant le saut. Cette hypothèse garantie, dans de futures études, l’exploration la cinématique du saut chez les fourmis et autres insectes. Translated to French by Jules Chabain (chabain2@illinois.edu)

Chinese:上翘腹部对破坏巨眼蚁 *Gigantiops destructor* （膜翅目，蚁科）跳跃表现与平衡的影响 跳跃是一种重要的运动形式, 动物们有多种机制来提升跳跃的表现。虽然跳跃行为在昆虫中很普遍, 但蚂蚁跳跃行为极为罕见。其中一个特例是南美洲的破坏巨眼蚁*Gigantiops destructor*, 其以跳跃捕食或避难而著称。该蚂蚁在起跳时会上翘腹部, 这一特殊行为引起了实验团队的注意。我们提出假说, 其上翘腹部的功能可能有二：提供起跳的推动力, 或在起跳腾空时维持躯体的平衡。我们利用高速摄像技术记录了破坏巨眼蚁在两个平台间跳跃的行为。之后, 我们对蚂蚁进行麻醉, 并用胶水限制其腹部的行动灵活性, 使其在之后的跳跃过程中不能上翘腹部, 并拍摄记录了其受限的跳跃行为。我们的实验结果显示, 用胶水限制腹部上翘显著降低了破坏巨眼蚁跳跃的最大水平距离、最大高度以及起跳速度, 进而支持了上翘腹部为其起跳提供推动力的假说。 虽然上翘腹部的行为受到限制, 蚂蚁在空中扭转时仍能够保持躯体平衡, 并可观察到蚂蚁腾空时, 腿部的扭转角度也发生了变化。这一现象说明, 腹部上翘被限制导致起跳过程中重心改变, 为了保持在空中的稳定性, 实验蚂蚁可能通过调整腿部运动来保持腾空中的身体平衡。我们的假说对后续其他蚂蚁物种及其他昆虫的跳跃行为的动力学研究有启示作用。Translated to Chinese by Dajia Ye (dajiaye@sas.upenn.edu)

Portuguese: Efeitos de Rotação Abdominal no Desempenho de Salto na Formiga *Gigantiops destructor* (Hymenoptera, Formicidae) O salto é uma forma importante de locomoção, e os animais empregam uma variedade de mecanismos para aumentar a performance de salto. Embora o salto seja comum nos insetos em geral, a capacidade de saltar é rara entre as formigas. Uma exceção é a formiga neotropical *Gigantiops destructor* (Fabricius 1804), conhecido por saltar para capturar presas ou escapar de ameaças. Notavelmente, essa formiga começa um salto girando seu abdômen para a frente enquanto sai do chão. Testamos as hipóteses de que a rotação abdominal é usada para fornecer impulso durante a saída do chão ou para estabilizar o momento de rotação durante a fase inicial do salto no ar. Utilizamos videografia de alta velocidade para caracterizar o desempenho de saltos de formigas operárias de G. destructor saltando entre duas plataformas. Em seguida, anestesiamos as formigas e aplicamos cola para impedir que o abdômen gire durante os saltos subsequentes, caracterizando novamente o desempenho do salto após restringir o abdômen dessa maneira. Nossos resultados suportam a hipótese de que a rotação abdominal fornece impulso adicional, pois a distância máxima, a altura máxima e a velocidade de saída dos saltos foram reduzidas pela restrição do movimento do abdômen, em comparação aos saltos das formigas não manipuladas e de controle. Em contraste, a estabilidade rotacional das formigas no ar não pareceu ser afetada. Alterações nos movimentos das pernas no ar das formigas restringidas sugerem que a estabilidade pode ser mantida usando as pernas para compensar as mudanças na distribuição da massa durante os saltos. Essa hipótese merece investigação em estudos futuros sobre a cinemática do salto de formigas ou outros insetos.Translated to Portuguese by Diego Vaz (dbistonvaz@vims.edu)

## Introduction

Locomotion is a fundamental ability of many animals and can take on a variety of forms including sliding, swimming, flying, walking, running, crawling, and jumping. Jumping specifically provides several important functions for animals, allowing them to navigate through complex environments, escape predation, or swiftly capture prey (Porro et al. 2017). Predator–prey interactions often select for high-performance jumping mechanisms in species which are able to jump. The ability to jump faster and farther can increase the chance of escape from predators, as in the high-speed leaps of Merriam’s kangaroo rats to escape from rattlesnakes strikes (Higham et al. 2017), or the chance of successfully capturing prey, as in jumping spiders (Chen et al. 2013). However, as the speed and distance of jumps increase, landing stances tend to become more unpredictable without corrections after takeoff, making it difficult for the animal to land in a proper orientation.

Mechanisms for stabilization and balance have arisen in many groups of jumping animals, allowing them to orient their bodies and control their trajectory to land properly. Species often use their tails or bend their abdomens to stabilize their body rotation during the course of a jump by repositioning their center of mass, preventing stochastic tumbling during a leap (Gillis et al. 2009; Cofer et al. 2010). Even more unique mechanisms have been found in jumping insects, such as the cuticular gears on the hind trochanters of planthoppers in the genus *Issus* which assure that both legs move as a single unit during takeoff, preventing the insect from jumping haphazardly to one side (Burrows and Sutton 2013). Insects are also well known for adaptations that increase acceleration and force while jumping. In addition to enlarged hindlegs, insects also employ power-amplification mechanisms, such as latches or stored elastic strain energy (Bennet-Clark and Lucey 1967; Bennet-Clark 1975; Burrows 2006; Patek et al. 2006) or generate thrust by moving body segments forward at take-off (Burrows and Morris 2002).

Jumping behavior in ants is quite rare; only 6 of the approximately 300 recognized genera of ants are known to contain species capable of jumping (Sorger 2015). These jumps can be broadly categorized into two groups (Wheeler 1922). The first are trap-jaw ants, including the genera *Odontomachus*, *Anochetus*, and *Strumigenys*, who use their spring-loaded mandibles to generate enough force to jump backwards—a behavior referred to as retrosalience (Wheeler 1922; Ali et al. 1992; Patek et al. 2006). Ants in the second group jump forward using their legs, and include the genera *Harpegnathos*, *Gigantiops*, *Myrmecia*, and some *Odontomachus* (Wheeler 1922; Sorger 2015). Despite the diversity of jumping behavior in ants, we know very little about jumping kinematics or how ants stabilize their body rotation during a jump to control trajectory and landing.

**Fig. 1:**
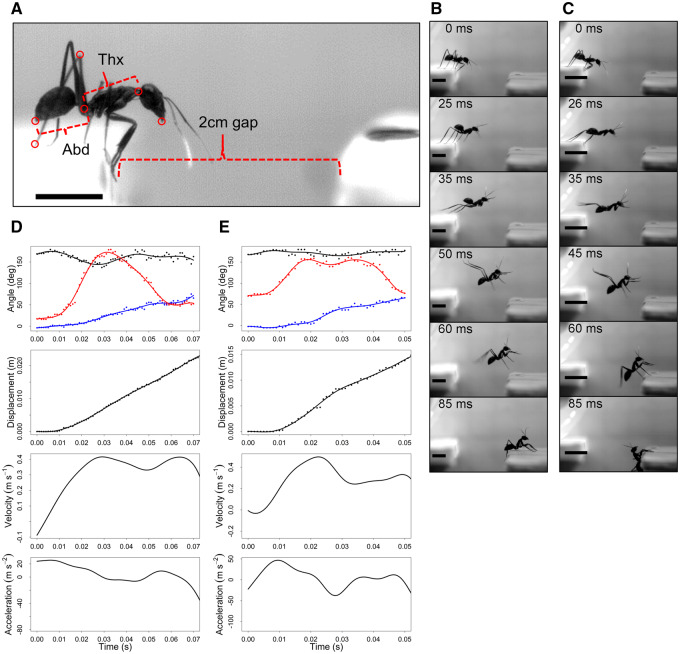
A) Frame taken from a video of *Gigantiops destructor* prior to jumping showing the arrangement of the two platforms that the ants jumped between in this study, as well as the segments we refer to as the abdomen (Abd) and thorax (Thx). The points on the body of the ant that were tracked throughout each video are shown as red circles. Scale bar=5 mm. **B**) Representative frames from a video of *G. destructor* jumping prior to experimentally restraining the abdomen. Scale bars=5 mm. **C**) Representative frames from a video of the same ant shown in Fig. 1B jumping after its abdominal movements were restricted using glue. Scale bars=5 mm. **D**) from top to bottom: angular position of the body (blue), legs (red), and abdomen (black) versus time over the course of the jump depicted in Fig. 1B. Points represent the angles calculated from each frame while curves show the spline functions fitted to each set of points; linear displacement versus time. Points represent the angles calculated from each frame while curves show the spline functions fitted to each set of points; velocity versus time function calculated by taking the derivative of the spline function shown in the displacement versus time panel; acceleration versus time calculated by taking the second derivative of the displacement versus time function. **E**) The same information depicted in Fig. 1D is shown for the jump depicted in Fig. 1C.

A well-known example of a leg propelled jumping ant is *Gigantiops destructor* (Fabricius 1804), which jumps to navigate through leaf litter, hunt arthropod prey, and escape predators (Wheeler 1922). *Gigantiops destructor* can jump 3–4 cm horizontally and reach maximum takeoff velocities of around 0.7 m/s over the course of a jump (Tautz et al. 1994). At the beginning of their jumps, workers will usually raise their abdomen (defined here as the abdominal segments posterior to the petiole, e.g., the gaster; Fig. 1A) during initial takeoff, and subsequently raise their hind legs above their thorax (defined here as the segments of the mesosoma anterior to the petiole; Fig. 1A) until the femur is almost perpendicular with the thorax while airborne (Tautz et al. 1994). Compared with other jumping ant species, this abdominal rotation during the initial stage of *G. destructor*’s jump is unusual (Wheeler 1922; Urbani et al. 1994; Sorger 2015). These movements resemble behaviors seen in other jumping arthropods that are known to be responsible for stabilizing or directing rotational momentum during jumps to ensure precise landings (Chen et al. 2013; Burrows et al. 2015). *Gigantiops destructor* is unique among jumping ants in that the center of mass is at the petiole, while the center mass of other ants is located more anteriorly (Tautz et al. 1994). This suggests that the heavier abdomens of *G. destructor* may transfer the center mass through the point of rotation during jumps to promote thrust.

We investigated the role of this abdominal movement on jumping performance of *G. destructor*. Specifically, we tested two non-mutually exclusive hypotheses: 1) Abdominal movement stabilizes body rotation by repositioning the ant’s center of mass during a jump, allowing for a more precise landing stance. 2) Abdominal movement provides thrust at the initial stages of the jump, allowing the ant to jump faster and farther. To test these hypotheses, we used a high-speed camera to film ants jumping between two platforms. We then experimentally restrained the ants’ abdomens using glue to prevent movement during future jumps. We predicted that if the function of abdomen movement during jumps is stabilization, then restraining the movement of the abdomen will influence the rotation of the ant’s body while jumping. Similarly, if the function of abdomen raising is to provide thrust for takeoff, then the trajectories and the maximum velocity of the jumps will decrease by restraining the abdomen.

## Materials and methods

### Colony collection and maintenance

A queenless colony of *G.destructor* with over 50 workers was collected from Nouragures National Nature Reserve, French Guiana (3.982411°S 52.563872°W ± 1 km) in March of 2016. Two additional colonies, one queenless and consisting of approximately 30 workers and brood and the second queenright with approximately 50 workers and brood were collected from the rainforest near ACTS research station in the Maynas province of Peru (3°14′60.00″S 72°54′36.00″W ± 1 km) in July of 2018. After collection the ant colonies were exported to the University of Illinois at Urbana–Champaign and housed in 17× 12×6 cm^3^ plastic containers with mesh vents added to the lids for airflow. All colonies were provided a 9 cm diameter petri dish that was partially filled with dental plaster to serve as artificial nests. The colonies were provided cotton stopped tubes containing water and a 20% sugar–water solution *ad libitum*, and fed chopped crickets twice a week. The enclosures were kept in a USDA-APHIS-PPQ certified quarantine facility that was set at 24 °C and 30–60% relative humidity and kept on a 12-h light cycle over the course of this study.

### Experimental design

Seventeen *G. destructor* workers from the three colonies were coerced to jump between two particle board platforms separated by a 2 cm gap by gently easing the ants toward the gap using a small petri dish (Supplementary Movie S1). All ants were first filmed jumping across the gap between 4 and 11 times each. Each ant was then randomly assigned to one of three groups: 1) the experimental group in which the ants were sedated and then had their abdomens restrained with glue; 2) a glue control group in which the ants were sedated and a drop of glue or enamel paint equivalent in mass to the amount of glue used in the experimental group was applied to the abdomen slightly posterior to the petiole’s junction with the thorax such that the abdomen was still able to move; and 3) a sedation control group in which the ants were sedated in the same manner as the experimental group but did not have their abdomens’ restrained. Two ants experienced all three treatments, with jumps recorded for the sedation control, glue control, and experimental treatment groups in that order.

After initial filming, ants in the experimental group (*n* = 8) were sedated by being placed inside a small petri dish held over ice until they stopped moving (approximately 1 min). Each ant was then transferred to a small Styrofoam board, where their heads, hind legs, and abdomen were held in place using crossed size 2 insect pins. A small drop of Titebond hide glue (Franklin International, Columbus, OH) was placed across the dorsal surface of the petiole and the Styrofoam board was placed over ice for 30 min until the glue dried. Afterward the insect pins were removed and the ant was returned to the petri dish at room temperature to recover. Ants in the glue control group (*n* = 7) were sedated in the same manner and had a drop of hide glue or enamel paint (Testor Corporation, Rockford, IL) added to the first gastral tergite (abdominal segment 3) so that the abdomen could still rotate. When paint was used, we were careful to measure out an amount approximately equivalent in mass to a drop of glue (Welsh two sample *t*-test, 10 drops of glue versus 10 drops of paint, *t* = 0.15, df = 17.4, *P* = 0.88). Ants in the sedation control group (*n* = 6) were anesthetized on ice and restrained for 30 min in the same manner as ants in the experimental group, but no glue was added. Ants in all groups were allowed to recover for at least 20 min after being sedated and having glue applied, and were subsequently filmed jumping across the 2 cm gap an additional four to nine times.

### Filming and video analysis

All jumps were filmed using a FastCam-X1280PCI high speed camera (Photron, USA Inc., San Diego, CA) set at 1000 frames/s. For each video, we used a custom MATLAB (MathWorks 2014) tracking script from Hedrick (2008) to track movement of the abdomen tip, petiole–thorax joint, hind tibia–femur joint, hind tarsal tip, dorsal anterior lip of the thorax, and the tip of the mandibles on each video frame (Fig. 1A). Only one leg was tracked in each video. The angle of the abdomen with respect to the thorax (*θ*) was calculated for each frame in Excel (Microsoft 2013) using the law of cosine:

(1) $$\theta =\cos^{-1}\left(\frac{p^{2}+q^{2}-r^{2}}{2pq}\right),$$

where *p* is the length of the thorax, *q* is the length of the abdomen, and *r* is the distance between the abdomen tip and the dorsal anterior tip of the thorax. Similarly, the angle between the tibia and femur (*α*) was calculated for each frame using the equation:

(2) $$\alpha =\cos^{-1}\left(\frac{z^{2}+v^{2}-w^{2}}{2zv}\right),$$

where *z* is the length of the tibia, *v* is the length of the femur, and *w* is the distance between the base of the femur and distal tip of the tibia. The angle of the body with respect to the horizontal (*β*) was calculated using the equation:

(3) $$\beta =\sin^{-1}\frac{s}{p},$$

where *s* is the length of the line drawn through the dorsal anterior tip of the thorax that is perpendicular to a horizontal line through the petiole. The change of all three angles over time was plotted using the function *smooth.Pspline* in the package *Pspline* in R (R Core Team 2018) by fitting a fourth order polynomial to each set of points with 10 degrees of freedom. This was visually determined to be the best method to optimize fit to the raw data while simultaneously producing a sufficiently smooth curve. For all three angles, the maximum angle was calculated using the newly created polynomials and the starting angle subtracted from this value to determine the net rotation experienced by each at this maximum angle.

Maximum velocity, horizontal distance, and maximum height of each jump were calculated using the movement of the dorso-anterior lip of the thorax as a common reference point with a custom script in R (R Core Team 2018). This script utilizes the *poly* function to fit a quadratic equation to each jump trajectory, after they were standardized so that the ants’ starting position begin at the origin, in the form of:

(4) $$y=ax^{2}+\mathrm{bx}+c,$$

where *y* and *x* are the vertical and horizontal position of the ant, respectively, and *a*, *b*, and *c* are coefficients. This equation is then used to calculate maximum height and horizontal distance attained over the course of the jump using the equations:

(5) $$h=\frac{-a}{2b},$$

where *h* is the maximum height, and

(6) $$x=\frac{-b\;\pm \;\sqrt{b^{2}-4ac}}{2a},$$

where *x* is the *x*-intercepts of the trajectory. The greater positive *x*-intercept value was used as the maximum horizontal distance traveled; the other intercept was always zero as the trajectories were standardized to begin at the origin. This value is not the actual horizontal distance traveled, but we consider this measurement more realistic for our analyses as whether the ants missed the platform would influence the actual horizontal distance traveled.

A displacement versus time function was generated for each jump using the *smooth.Pspline* function by applying a fourth order polynomial to the data with 10 degrees of freedom. The first and second derivatives of this function were used to create velocity versus time and acceleration versus time graphs. Takeoff velocity and acceleration were both calculated using these functions.

### Statistical analyses

All statistics were performed in R (R Core Team 2018). Takeoff velocity, maximum acceleration, takeoff angle, height, horizontal distance, body rotation, and leg rotation were compared for jumps before and after applying treatment conditions within each of the three experimental groups. This was done by creating a linear mixed effect model within each experimental group treating individual ant identity as a random effect using the *lmer* function in the *lme4* package in R. These models were then compared with a null model consisting of an intercept and individual ant identity as a random effect with an ANOVA using the *anova* function in the *car* package in R. We assessed significance at *P* < 0.006 based on a Bonferroni correction for eight comparisons (e.g., each variable) (Rice 1989). The relationships between net leg movement angle and net body rotation, net abdominal movement and maximum velocity, net abdominal movement and net body rotation, and net leg movement and maximum velocity were compared with a linear regression analysis using the *lm* function.

## Results

### Unrestrained jump kinematics

Unrestrained ant jumps occurred over 73.8 ± 11.6 ms (mean ± SE), with jump trajectories covering a horizontal distance of 2.2 ± 0.7 cm and reaching a maximum height of 0.49 ± 0.24 cm (Fig. 1B andTable 1). Ants reached an average maximum velocity of 0.47 ± 0.09 m/s, with the fastest ant reaching a max velocity of 0.80 m/s. Ants obtained an average maximum acceleration of 31.1 ± 11.0 m/s^2^ prior to takeoff, with a maximum of 74.1 m/s^2^ (Table 1). Representative displacement versus time, velocity versus time, and acceleration versus time graphs are shown in Fig. 1D.

**Table 1:** Mean (±SE) values for each of eight variables measured before and after experimental manipulation (abdomen restrained, glue control, or sedation control), and summary of the linear mixed effect models for each jump performance variable and treatment group

Variable	Manipulation	Pre-manipulation mean±SD (*N*, *n*)	Post-manipulation mean±SD (*N*, *n*)	Linear mixed effect model		ANOVA of model vs. null	
				Difference of means±SD	*t*-Value	Chi square-value	*P*-value
Abdomen rotation (°)	Abdomen restrained	27.7±15.2 (8, 54)	2.4±6.5 (8, 53)	−23.6±2.2	−10.9	80.8	<0.0001*
	Glue control	32.8±12.1 (7, 41)	26.9±14.7 (7, 37)	−6.2±2.8	−2.2	4.8	0.03
	Sedation control	27.0±14.3 (6, 38)	37.0±12.1 (6, 34)	10.3±2.9	3.6	11.6	0.0006*
Takeoff velocity (m/s)	Abdomen restrained	0.51±0.11 (8, 54)	0.46±0.12 (8, 53)	−0.05±0.02	−3.1	9.4	0.002*
	Glue control	0.42±0.06 (7, 41)	0.41±0.05 (7, 37)	−0.005±0.01	−0.4	0.15	0.70
	Sedation control	0.43±0.06 (6, 38)	0.44±0.07 (6, 34)	0.01±0.01	0.7	0.54	0.46
Acceleration (m/s^2^)	Abdomen restrained	35.3±12.8 (8, 54)	35.6±21.0 (8, 53)	0.29±3.4	0.09	0.008	0.93
	Glue control	28.1±8.9 (7, 41)	24.2±6.6 (7, 37)	−3.9±1.8	−2.2	4.8	0.03
	Sedation control	28.0±8.5 (6, 38)	28.8±20.7 (6, 34)	0.9±3.6	0.3	0.06	0.81
Takeoff angle (°)	Abdomen restrained	38.8±11.4 (8, 54)	37.5±13.4 (8, 53)	−1.2±2.4	−0.5	0.27	0.61
	Glue control	39.7±10.0 (7, 41)	32.8±14.7 (7, 37)	−6.8±2.6	−2.6	6.5	0.01
	Sedation control	38.2±12.9 (6, 38)	35.0±16.0 (6, 34)	−2.2±2.7	−0.8	0.7	0.40
Height (cm)	Abdomen restrained	0.51±0.28 (8, 54)	0.27±0.16 (8, 53)	−0.24±0.04	−5.5	27.2	<0.0001*
	Glue control	0.42±0.21 (7, 41)	0.30±0.16 (7, 37)	−0.12±0.04	−3.0	8.6	0.003*
	Sedation control	0.38±0.23 (6, 38)	0.34±0.26 (6, 34)	−0.03±0.04	−0.6	0.4	0.53
Horizontal distance (cm)	Abdomen restrained	2.4±0.9 (8, 54)	1.3±0.7 (8, 53)	−1.13±0.14	−8.0	50.5	<0.0001*
	Glue control	1.8±0.6 (7, 41)	1.6±0.6 (7, 37)	−0.28±0.09	−3.2	8.7	0.003*
	Sedation control	1.7±0.6 (6, 38)	1.6±0.7 (6, 34)	−0.09±0.1	−0.7	0.5	0.48
Body rotation (°)	Abdomen restrained	58.0±18.7 (8, 54)	58.8±23.4 (8, 53)	−0.4±4.0	−0.1	0.005	0.94
	Glue control	56.4±14.2 (7, 41)	62.5±21.9 (7, 37)	6.1±4.0	1.5	2.3	0.13
	Sedation control	53.5±14.2 (6, 38)	55.6±20.0 (6, 34)	2.1±4.0	0.5	0.3	0.60
Leg movement (°)	Abdomen restrained	115.7±30.7 (8, 54)	101.5±29.1 (8, 53)	−14.0±5.6	−2.5	6.1	0.01
	Glue control	112.7±21.1 (7, 41)	110.9±29.4 (7, 37)	−1.4±5.4	−0.3	0.07	0.79
	Sedation control	111.4±24.5 (6, 38)	111.5±23.3 (6, 34)	0.2±5.4	0.03	0.001	0.97

Models that are significantly different from their respective null models with a Bonferroni adjusted *P*-value of <0.006 for eight comparisons (e.g., each variable) are marked with an asterisk. *N*=number of ants in each group, *n*=total number of jumps filmed.

**Fig. 2:**
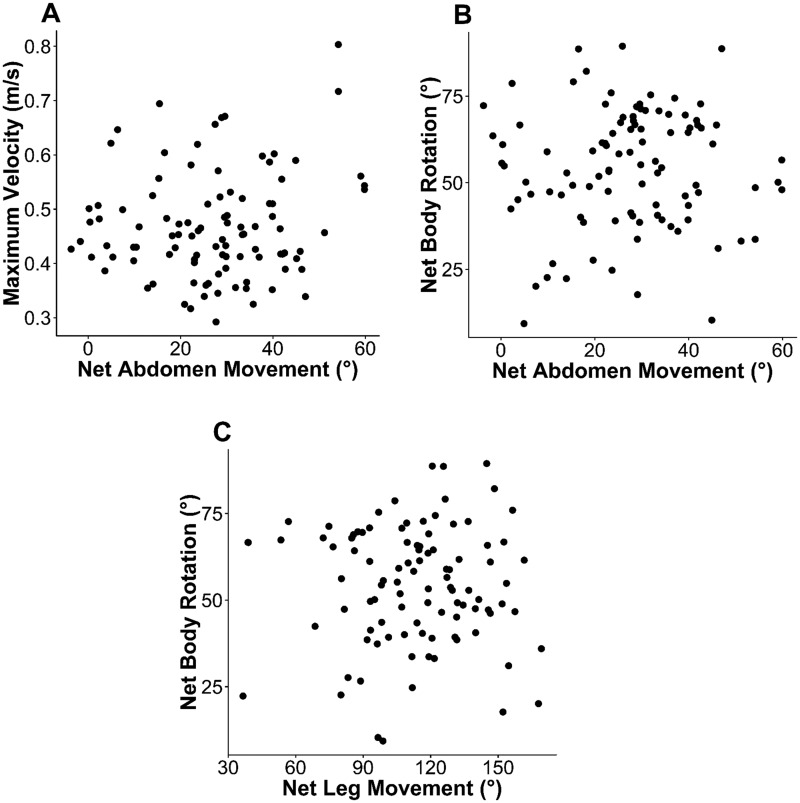
Scatterplots depicting relationships between abdominal movement, velocity, and body rotation. Each dot represents data from one unrestrained jump of *G. destructor*. Data are pooled for all individuals. No relationship was found to be significant (Pearson’s regression, df = 50, *P* > 0.05). **A**) Takeoff velocity versus net abdomen movement, defined as the maximum change in abdomen angle with respect to the thorax over the course of the jump; **B**) net body rotation, defined as the maximum change in body angle with respect to the horizontal over the course of a jump, versus net abdomen movement; and **C**) net body rotation versus net leg movement, defined as the maximum change in angle of the tibia with respect to the femur over the course of a jump.

Comparing performance variables in unmanipulated jumps, we found no relationship between maximum velocity and abdominal rotation (Fig. 2A; linear regression; *F* = 0.87; df = 114; *P* = 0.35), abdominal rotation and body rotation (Fig. 2B; linear regression; *F* = 1.7; df = 114; *P* = 0.19), leg movement and body rotation (Fig. 2C; linear regression; *F* = 2.03; df = 114; *P* = 0.16), or leg movement and maximum velocity (linear regression; *F* = 0.15; df = 114; *P* = 0.70). We also found no relationship between the time at which the smallest angle between the abdomen and thorax was reached (*T*_abd_) and the time at which the ant reached maximum velocity (linear regression; *F* = 0.31, df = 114, *P* = 0.58), or *T*_abd_ and the time at which the body reached its greatest angle with respect to the horizontal (linear regression; *F* = 0.08, df = 114, *P* = 0.93), There was, however, a significant positive relationship between *T*_abd_ and the time at which the legs were most extended (linear regression, *R*^2^ = 0.12, *F* = 15.6, df = 114, *P* = 0.0001).

### Effectiveness of glue in restraining abdominal movement

**Fig. 3:**
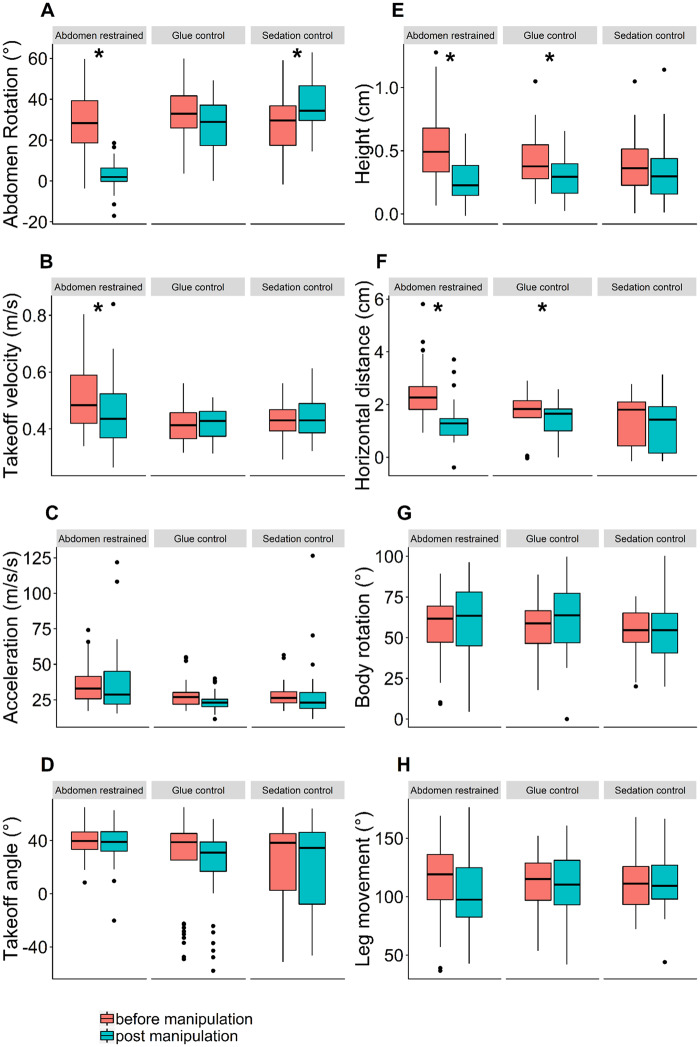
Boxplots summarizing jump performance metrics for each experimental group. In each facet the left boxplot (red) depicts unmanipulated jumps, while the right boxplot (blue) depicts jumps post-manipulation. **A**) Abdomen rotation; **B**) takeoff velocity; **C**) acceleration; **D**) takeoff angle; **E**) maximum height; **F**) horizontal distance traveled; **G**) body rotation with respect to the horizontal; **H**) movement of the tibia with respect to the femur. Detailed statistical analyses for these comparisons are shown in Table 1. Models that are significantly different from their respective null models with a Bonferroni adjusted *P*-value of <0.006 for eight comparisons (e.g., each variable) are marked with an asterisk.

Our experimental treatment successfully limited the movement of the ants’ abdomens during jumps (Figs. 1C, E and 3A); abdominal rotation in ants in the experimental group was reduced by 91% of the value pre-manipulation, lowering from 27.7 ± 15.2° pre-manipulation to 2.4 ± 6.5° post-manipulation (Table 1). Ants in the glue control group also showed a slight reduction of ∼18% in abdominal rotation, lowering from 32.8 ± 12.1° pre-manipulation to 26.9 ± 14.7° post-manipulation, but this reduction was not statistically significant after Bonferroni correction (Fig. 3A andTable 1). The sedation control group experienced an increase in abdominal rotation after treatment from 27.0 ± 14.3° pre-manipulation to 37.0 ± 12.1° post-manipulation, an increase of ∼30% (Fig. 3A andTable 1).

### Effects of abdominal movement on speed and trajectory of jumps

Restricting abdominal movement decreased takeoff velocity by ∼10% compared with unmanipulated jumps, decreasing from 0.51 ± 0.11 m/s pre-manipulation to 0.46 ± 0.12 m/s post-manipulation; a similar reduction in performance was not seen in either control group (Fig. 3B andTable 1). Acceleration during takeoff did not differ in any of the three groups after manipulation (Fig. 3C andTable 1). Restraining abdominal movement did not change the takeoff angle of jumps; a slight reduction of 17% from 39.7 ± 10.0° pre-manipulation to 32.8 ± 14.7° post-manipulation did occur in the glue control group, but this difference was not statistically significant after Bonferroni correction (Fig. 3D andTable 1). Maximum jump height decreased after restraining the abdomen in the experimental group, changing by ∼47% from 0.51 ± 0.28 cm pre-manipulation to 0.27 ± 0.16 cm post-manipulation. The glue control group also experienced a 28% reduction in jump height from 0.42 ± 0.21 cm pre-manipulation to 0.30 ± 0.16 cm post-manipulation (Fig. 3E andTable 1). The sedation control group did not experience any decrease in jump height post-manipulation (Fig. 3E andTable 1). Horizontal distance of jumps was likewise decreased in both the experimental group and the glue control group, decreasing 46% from 2.4 ± 0.9 cm pre-manipulation to 1.3 ± 0.7 cm post-manipulation in the experimental group and decreasing 11% from 1.8 ± 0.6 cm pre-manipulation to 1.6 ± 0.6 cm post-manipulation in the glue control group (Fig. 3F andTable 1). There was no decrease in horizontal jump distance in the sedation control group (Fig. 3F andTable 1).

Pre-manipulation takeoff velocities, accelerations, heights, and horizontal distances traveled were higher in the experimental group compared with the control groups, with the reduction in velocity post-manipulation resulting in takeoff velocities that are still higher than pre-manipulation takeoff velocities in either control group. Comparing ants across all treatment groups, we found that two ants in the experimental group with mean pre-manipulation jump velocities of 0.61 ± 0.09 and 0.62 ± 0.03 m/s could be classified as outliers (i.e., their mean velocities exceeded the overall mean pre-manipulation velocity by greater than 1.5 times the interquartile range [IQR]; Mean_Ant_ > Mean_Total_ + 1.5 * IQR), and were the cause of higher velocities seen in the experimental group relative to the control groups. Examining the videos and filming conditions of these two ants, we found no effective explanation as to why these ants jumped faster than the others, although they were among the biggest ants filmed, suggesting that body size may have played a role. Excluding these ants from statistical analyses resulted in takeoff velocity means of 0.45 ± 0.08 m/s pre-manipulation to 0.40 ± 0.08 m/s post-manipulation, which was both on-par with pre-manipulation velocities experienced by the control groups and lower than that of either control group post-manipulation. Pre-manipulation values for jump height and horizontal distance traveled were also closer to those experienced by the control groups after excluding these two ants from the dataset, although pre-manipulation acceleration remained inexplicably higher in the experimental group compared with the control groups. Excluding these ants from statistical analyses does not change the statistical significance of our results, only the pre-manipulation differences in velocity between the experimental and control groups (Supplementary Table S1).

### Effects of abdomen movement on body rotation and leg movement

There was no difference in body rotation in the experimental or glue control groups (Fig. 3G andTable 1). Leg rotation also did not in general differ pre- and post-manipulation although there was a slight reduction of 12% from 115.7 ± 30.7° pre-manipulation to 101.5 ± 29.1° post-manipulation in the experimental group; this decrease was not statistically significant after Bonferroni correction (Fig. 3H andTable 1).

## Discussion

We investigated the influence of abdominal rotation on jump performance in the ant *G. destructor*. Inhibiting abdominal movement decreased the maximum velocity, horizontal distance, and height of the ants’ jumps, but did not influence body rotation. Jump height and distance were also reduced to a lesser extent in ants where glue or paint was added to the abdomen but did not restrict its movement (glue control). This loss of performance in the glue control group likely resulted from the added weight shifting the center of mass of the ant, reducing the takeoff angle which the ant is able to jump at successfully while maintaining balance during the jump. This interpretation is supported by changes to takeoff angle which was also slightly reduced in the glue control group, but not in the experimental or sedation control groups. Our results therefore support the hypothesis that abdominal raising at the initial stage of *G. destructor* jumping functions to generate additional thrust during takeoff. This mechanism is not unique to *G. destructor* and has been shown to occur in previous research on other jumping animals, such as walking sticks and semi-terrrestrial tadpoles (Burrows and Morris 2002; Veeranagoudar et al. 2009). We also found that pre-manipulation measures of performance were higher in ants in the experimental group. This discrepancy was caused by two ants who had pre-manipulation takeoff velocities exceeding those of any other ants in the experimental or control groups. Excluding these ants from analyses did not change the results, but made the pre-manipulation jump performances of the experimental group on par with those in the control groups. These two individuals were among the largest ants we filmed. An examination of how body mass influences components of jump performance would be an interesting area for future work in this system.

We did not find that abdominal movement influenced rotational stability during jumps. Although our results did not support the stabilization hypothesis, previous research on other species of jumping insects provides evidence that the rotation of the abdomen remedies tumbling during jumping in those species (Cofer et al. 2010; Burrows et al. 2015). The functional difference of abdomen rotation in different species of jumping insects could be related to differences in abdomen shape. The abdomens of mantises and locusts are relatively thin and long, and they function similar to the tails of lizards to help with body balance and rotational stability during jumps (Cofer et al. 2010; Libby et al. 2012; Burrows et al. 2015). In contrast, the abdomen of *G. destructor* is relatively round and short, so the function of its abdomen could be less related to balance. In jumping insects with longer abdomens, small changes in abdomen angle result in large changes in the center of mass and rotational momentum (Cofer et al. 2010; Burrows et al. 2015).

Further experiments are needed to fully rule out of the role of abdominal movement in jumping stability in this ant. For example, body rotation could be occurring in a subtler manner than we were able to detect with our methods. Our understanding of jump mechanics in *G. destructor* may improve if we can analyze other kinematic changes during jumps, such as the position and movement of both legs and body rotation in three-dimensional space. For example, when the abdomen was glued in place, the net leg rotation slightly decreased compared with unrestrained ants. This result suggests that leg raising during the airborne phase of the jump could help with stabilization and offset rotational instability resulting from unrestrained abdominal rotation. This hypothesis is partially supported by our finding that the timing of maximum abdominal rotation and full leg extension are significantly correlated. Moving the abdomen up and forward at the initial stage of jumping likely moves the ant’s center of mass anteriorly, and the corresponding leg raising may prevent the body from rotating forward as a result. As there is likely no change in the center of mass during jumps where the abdomen is restrained, the corresponding leg rotation seen in unrestrained jumps becomes unnecessary. Previous research in other hind-leg jumping arthropods also exhibit similar leg movements while the insect is airborne (Bennet-Clark and Lucey 1967). Future work involving detailed models of changes in the center of mass during jumps is likely needed to discern these interactions more effectively.

Unrestrained jump kinematics from this experiment agree with previous research on *G. destructor*’s jumping ability that found that the maximum velocity over the course of a jump was 0.7 m/s and the horizontal distance traveled was 3–4 cm (Tautz 1994). We also found support for the hypothesis that abdominal movement during jumping behavior provides a mechanism to increase thrust for jumping, but not necessarily to stabilize body rotation during jumps. Changes in movement patterns of the ants’ legs in ants with restrained abdomens suggest that a lack of stability may be offset by compensatory leg movements. This research helps us understand the biomechanics and kinematics of ants jumping and will benefit from field observations that allow us to place these observations into a more ecological context.

## Supplementary Material

obz033_Supplementary_Data
