# Supplementary material for: Effects of Abdominal Rotation on Jump Performance in the Ant Gigantiops destructor (Hymenoptera, Formicidae)
Source: Integr Org Biol. 2019 Dec 18;2(1):obz033. doi: 10.1093/iob/obz033 (PMC7671114; doi:10.1093/iob/obz033)
Supplement: obz033_Supplementary_Data [file obz033_supplementary_data.zip › Gigantiops_Manuscript_FinalSubmission_SupplementalTable1.docx]

**Supplemental Table 1.**

| Variable | Manipulation | Pre-manipulation mean ± sd (N, n) | Post-manipulation mean ± sd (N, n) | Linear mixed effect model | | ANOVA of model vs. null | |
| --- | --- | --- | --- | --- | --- | --- | --- |
|  |  |  |  | Difference of  means ± sd | *t*-value | Chi Square-value | *p*-value |
| Abdomen rotation (°) | Abdomen restrained | 26.1 ± 15.2 (6, 37) | 1.5 ± 5.9 (6, 39) | -23.8 ± 2.5 | -9.5 | 60.5 | <0.0001* |
|  | Glue control | 32.8 ± 12.1 (7, 41) | 26.9 ± 14.7 (7, 37) | -6.2 ± 2.8 | -2.2 | 4.8 | 0.03 |
|  | sedation control | 27.0 ± 14.3 (6, 38) | 37.0 ± 12.1 (6, 34) | 10.3 ± 2.9 | 3.6 | 11.6 | 0.0006* |
| Takeoff velocity (m/s) | Abdomen restrained | 0.45 ± 0.08 (6, 37) | 0.40 ± 0.08 (6, 39) | -0.06 ± 0.02 | -3.5 | 11.2 | 0.0008* |
|  | Glue control | 0.42 ± 0.06 (7, 41) | 0.41 ± 0.05 (7, 37) | -0.005 ± 0.01 | -0.4 | 0.15 | 0.70 |
|  | sedation control | 0.43 ± 0.06 (6, 38) | 0.44 ± 0.07 (6, 34) | 0.01 ± 0.01 | 0.7 | 0.54 | 0.46 |
| Acceleration (m/s^2^) | Abdomen restrained | 34.4 ± 14.4 (6, 37) | 35.2 ± 19.2 (6, 39) | 0.44 ± 3.9 | 0.11 | 0.02 | 0.88 |
|  | Glue control | 28.1 ± 8.9 (7, 41) | 24.2 ± 6.6 (7, 37) | -3.9 ± 1.8 | -2.2 | 4.8 | 0.03 |
|  | sedation control | 28.0 ± 8.5 (6, 38) | 28.8 ± 20.7 (6, 34) | 0.9 ± 3.6 | 0.3 | 0.06 | 0.81 |
| Takeoff angle (°) | Abdomen restrained | 41.2 ± 11.5 (6, 37) | 39.8 ± 10.1 (6, 39) | -1.3 ± 2.5 | -0.5 | 0.33 | 0.56 |
|  | Glue control | 39.7 ± 10.0 (7, 41) | 32.8 ± 14.7 (7, 37) | -6.8 ± 2.6 | -2.6 | 6.5 | 0.01 |
|  | sedation control | 38.2 ± 12.9 (6, 38) | 35.0 ± 16.0 (6, 34) | -2.2 ± 2.7 | -0.8 | 0.7 | 0.40 |
| Height (cm) | Abdomen restrained | 0.49 ± 0.25 (6, 37) | 0.25 ± 0.14 (6, 39) | -0.24 ± 0.05 | -5.1 | 23.2 | <0.0001* |
|  | Glue control | 0.42 ± 0.21 (7, 41) | 0.30 ± 0.16 (7, 37) | -0.12 ± 0.04 | -3.0 | 8.6 | 0.003* |
|  | sedation control | 0.38 ± 0.23 (6, 38) | 0.34 ± 0.26 (6, 34) | -0.03 ± 0.04 | -0.6 | 0.4 | 0.53 |
| Horizontal distance (cm) | Abdomen restrained | 2.0 ± 0.5 (6, 37) | 1.2 ± 0.4 (6, 37) | -0.88 ± 0.1 | -8.6 | 52.7 | <0.0001* |
|  | Glue control | 1.8 ± 0.6 (7, 41) | 1.6 ± 0.6 (7, 37) | -0.28 ± 0.09 | -3.2 | 8.7 | 0.003* |
|  | sedation control | 1.7 ± 0.6 (6, 38) | 1.6 ± 0.7 (6, 34) | -0.09 ± 0.1 | -0.7 | 0.5 | 0.48 |
| Body rotation (°) | Abdomen restrained | 57.9 ± 15.4 (6, 37) | 66.3 ± 17.7 (6, 39) | 7.7 ± 3.7 | 2.1 | 4.4 | 0.04 |
|  | Glue control | 56.4 ± 14.2 (7, 41) | 62.5 ± 21.9 (7, 37) | 6.1 ± 4.0 | 1.5 | 2.3 | 0.13 |
|  | sedation control | 53.5 ± 14.2 (6, 38) | 55.6 ± 20.0 (6, 34) | 2.1 ± 4.0 | 0.5 | 0.3 | 0.60 |
| Leg movement (°) | Abdomen restrained | 111.9 ± 30.5 (6, 37) | 96.5 ± 30.5 (6, 39) | -16.1 ± 6.4 | -2.5 | 6.1 | 0.01 |
|  | Glue control | 112.7 ± 21.1 (7, 41) | 110.9 ± 29.4 (7, 37) | -1.4 ± 5.4 | -0.3 | 0.07 | 0.79 |
|  | sedation control | 111.4 ± 24.5 (6, 38) | 111.5 ± 23.3 (6, 34) | 0.2 ± 5.4 | 0.03 | 0.001 | 0.97 |
